# Volatile organic compounds of camel milk and shubat across Kazakhstan's regions, seasons, and breeds

**DOI:** 10.1016/j.heliyon.2024.e35365

**Published:** 2024-07-29

**Authors:** Zauresh Bilal, Askar Kondybayev, Aikerim Ospanova, Helene Tormo, Shynar Akhmetsadykova, Farida Amutova, Bernard Faye, Gaukhar Konuspayeva

**Affiliations:** a Al-Farabi Kazakh National University, Biotechnology Department, 71 Al-Farabi Avenue, 050040, Almaty, Kazakhstan; b LLP "Scientific and Production Enterprise "Antigen", 4, Azerbayeva Str, Almaty Region, 040905, Kazakhstan; c M. Auezov South Kazakhstan University, Shymkent, Kazakhstan; d Departement Sciences de L’Agroalimentaire et de La Nutrition INP-EI Purpan, Université de Toulouse, 75, Voie Du TOEC, BP 57611, F-31076, Toulouse, Cedex 3, France; e LLP «Kazakh Research Institute for Livestock and Fodder Production», Horse and Camel Breeding Department, 51, Zhandosov Str., Almaty, 50035, Kazakhstan; f Center of International Cooperation on Agriculture Research for Development – CIRAD, UMR SELMET, Campus International de Baillarguet, 34398, Montpellier, Cedex 5, France

**Keywords:** Camel milk, *Shubat*, Volatile organic compounds, Camel species, Variability, Kazakhstan

## Abstract

Despite extensive research in recent years on camel milk composition and health benefits, limited scientific data exists on the volatile organic compound profiles of camel milk and its fermented product, *shubat*. This study analyzed volatile organic compounds (VOCs) in raw camel milk and *shubat* from six Kazakh farms across all seasons. We found that camel milk displayed higher concentrations of aldehydes, ketones, and alcohols with the main two compounds in milk being acetone and (2-Aziridinylethyl) amine. Conversely, the majority of volatile organic compounds in *shubat* samples belonged to esters, but the predominant compounds by concentration were ethanol, dimethylamine, propanoic acid, and octanoic acid. Seasonality emerged as the primary driver of variation in milk, with heptanal being the most discriminative compound. Fermented milk showcased regional diversity likely driven by distinct microbial communities. Findings demonstrate the dynamic nature of camel milk's aromatic properties, which are influenced by multiple factors that contribute to its distinctive sensory characteristics.

## Highlights

• First comprehensive analysis of VOC profiles in camel milk and shubat.
• Seasonal forage differences influence camel milk composition and aroma profiles.
• Shubat's VOC profile varies by region, indicating regional microbiota differences.

## 1 Introduction

Raw camel milk and its fermented form (*shubat*) are integral parts of diet and livelihood of camel-rearing communities in arid and semi-arid regions, providing sustenance, hydration, and important nutrients in challenging environments [1]. They are a valuable source of nutrients and have gained significant attention for their potential health benefits. Although camel milk shares a general composition with cow milk, the specific components in their compositions differ significantly as for example the lack of β-lactoglobulin, the presence of two specific proteins (WAP and PGRP), the particular richness in vitamin C and the fatty acid composition [2]. Camel milk has been associated with several health benefits, including improved immune function, gastrointestinal health, and potential anti-diabetic properties [3].

The process of fermenting camel milk into *shubat* is a cultural and dietary tradition among camel-rearing communities of Central Asia, particularly in Kazakhstan [4]. In the traditional process of making *shubat* in Kazakhstan, fresh camel milk is fermented with a small amount of previously soured milk in a special container for 1–2 days, with regular mixing during fermentation. This process systematically categorizes *shubat* into three acidity levels—weak, medium, and strong—based on variations in fermentation duration. Notably, key lactic acid bacteria species, including *Lactobacillus helveticus*, *Lactobacillus delbrueckii* subsp. *bulgaricus*, and *Streptococcus thermophilus*, have been identified as pivotal in this fermentation process [5].

Despite extensive research over the past 15 years on camel milk composition and health benefits, a significant gap persists in our understanding of the aromatic profiles of camel milk and its fermented product, *shubat*. Aroma is a crucial sensory attribute that greatly affects consumer acceptance. In a comparative context with fresh cow's milk, camel milk has been rated lower for taste, aroma, and overall acceptance [6]. The current reliance on subjective descriptions of camel milk aroma limits our ability to accurately compare samples, identify key contributors, and understand the role of influencing factors. Milk volatile organic compounds (VOCs) are important contributors to the flavor and aroma of milk and dairy products. While some research has explored the VOCs in fermented camel milk and cheese products [7,8], the aromatic profile of camel milk itself remains surprisingly less studied. Study of cheese made with camel milk revealed 40 VOCs, the majority of which belonged to volatile alcohols [7]. It is reasonable to expect that the aroma would vary with changes in the composition of camel milk. Numerous studies have reported significant variations in the composition of camel milk based on season [9], region and breed [10]. This study aims to report the VOC profiles of camel milk and *shubat* of Kazakhstan, as well as exploring how these profiles change in response to the aforementioned characteristics, namely region, season, and breed.

## 2 Materials and methods

### 2.1 Sampling procedure

Samples of raw camel milk and *shubat* produced at six farms were collected across 4 seasons of the calendar year in Kazakhstan. The farms were selected from three regions, namely Almaty, Kyzylorda and Turkistan regions. All farms produce commercial *shubat*, with daily outputs ranging from 30 to 150 L. The *shubat* is intended for sale in local and regional markets. As illustrated in Table 1, a total of 54 samples of raw milk and 20 samples of *shubat* were collected from each seasons, regions, and camel breeds. To ensure the preservation of quality, each milk sample was meticulously transported to the laboratory in specialized refrigerated containers, maintaining the highest possible cleanliness and sterility standards. The internal temperature of containers during transportation was maintained at a constant temperature of 4 ± 2 °C using additional refrigerants. pH measurements were taken immediately after milking on farms using a portable pH meter (HM Digital, USA- South Korea), which was calibrated daily in advance. Upon arrival at the laboratory, pH was measured again. The pH values of samples are presented in Supplementary Table S1. The *shubat* samples were intentionally collected with a higher pH (weak *shubat*), achieved by incorporating fresh camel milk, to prevent excessive fermentation (resulting in overly high acidity) during transportation. The final pH levels, assessed before analysis, fell within the acceptable ranges consistent with consumer preferences for either the "medium" or "strong" versions of *shubat*.

**Table 1:** Distribution of camel milk and shubat samples by seasons, regions, and camel breeds.

		Camel milk	Shubat
Seasons	Spring	14	4
	Summer	13	5
	Autumn	13	6
	Winter	14	5
Regions	Almaty	19	5
	Turkistan	15	8
	Kyzylorda	20	7
Breeds	Dromedary	22	–
	Bactrian	4	–
	Hybrid	14	–
	Mixed	14	20

### 2.2 Volatile organic compound analysis

Solid phase microextraction (SPME) technique with polydimethylsiloxane/divinylbenzene PDMS/DVB (65/10) extraction fiber (Agilent technologies, Switzerland) was used for detection of VOCs. The extraction was carried out at 60 °C for 30 min in 20 mL vial capped with magnetic Silicon/PTFE screw caps. Thereafter, the sampled fiber was placed for 3 min in GC injector, pre-heated to 260 °C, for desorption of VOCs in splitless mode. Separation of VOC was carried out using a 30m long capillary column HP-5MS (Agilent, USA), inner diameter - 0.25 mm and a film thickness of 0.25 μm at a constant gas carrier (helium grade A with a purity of 99.995 %, LLP "Dobromir", Kazakhstan) flow rate of 1.0 mL min^−1^.

Detection of VOCs in samples were performed using gas chromatograph with mass spectrometer 7890B/5977 B from Agilent Technologies, USA. The chromatograph was equipped with an autosampler MultiPurpose Sampler MPS (Gerstel, Germany, 2017), which allowed to automate the analysis of samples. The MassHunter GC/MS Acquisitions B.May 07, 2479 and Agilent MSD ChemStation software (version F.March 01, 2357) were used to control the gas chromatographic system, record and process the chromatographic data. Data processing included determining retention times, peak heights and areas, and processing of spectral information obtained with the mass spectrometric detector. To investigate the obtained mass spectra, the library Wiley 10th edition was used (the total number of spectra in the library was more than 550,000).

The chromatography temperature was programmed according to Pan et al. [11] with some modifications: from 40 °C (5 min) to 60 °C at a heating rate of 5 °C min^−1^ (3 min), then by heating to 120 °C (3 min) at 6 °C min^−1^ and followed by heating to 260 °C (0 min) at 10 °C min^−1^. The total chromatographic time was 34 min. The temperatures of the interface, ion source and quadrupole of the mass spectrometric detector were 260, 230 and 150 °C, respectively. Mass spectrometric detection was performed in the SCAN mode. The spectra were collected in a mass range of *m*/*z* 33–450.

Samples were analyzed in triplicate on gas chromatograph with mass spectrometer detector 7890 B/5977 B quadrupole, with electron impact ionization (Agilent, USA, 2017). Just before GC–MS analysis, 1 μL of the internal standard 3-heptanol (99 %; Sigma Aldrich, USA) (0.8 g L^−1^ in water) was applied to 1 mL of the samples. To calculate the VOC concentration in a sample, the peak area of the VOC was divided by the peak area of 3-Heptanol and then multiplied by the concentration of 3-Heptanol. Compound identification was attained using the NIST20 Mass Spectrometry library.

### 2.3 Statistical analyses

Milk and *shubat* were analyzed separately. The objectives of the statistical analyses were: (i) to explore the diversity of VOCs profile according the main factors (breed, region, season); (ii) to identify homogenous groups of samples regarding their VOCs profiles; (iii) to test the link between these groups and the potential explaining parameters, i.e., breed, region and season; (iv) to identify the main VOCs able to distinguish significantly the seasonal, regional and specific profiles. To achieve these different objectives, the following steps of the statistical strategy were taken.

(i) Principal component analysis – PCA (Pearson (n-1)) was applied on the tables including i samples (milk or *shubat*) and j VOCs with a representation of the variation factors (breed, region, season) as supplementary variables.
(ii) Agglomerative hierarchical clustering (AHC) following the PCA in order to identify homogenous groups of samples regarding their VOCs composition.
(iii) Contingency tables crossing the classes issued from AHC with the variation factors, their independence being assessed by Chi^2^-test.
(iv) Factorial discriminant analysis (ascending stepwise model) applied on the variation factors (breed, region, season) in order to identify the main discriminating VOCs and to evaluate the percentage of well-classed samples (i.e., to compare the number of samples for each breed, region or season, classified according to their VOC profiles and according to their effective belonging to such breed, region or season)

After the Discriminant analysis, a validation of the results based on the reiteration (10 times) on N rows of the data table (90 % of the table randomly selected) was achieved to test the robustness and the stability of the results. The software used for the analyses was XLStat 2016 (Addinsoft SARL, New York, NY).

## 3 Results

### 3.1 Camel milk

**Fig. 1:**
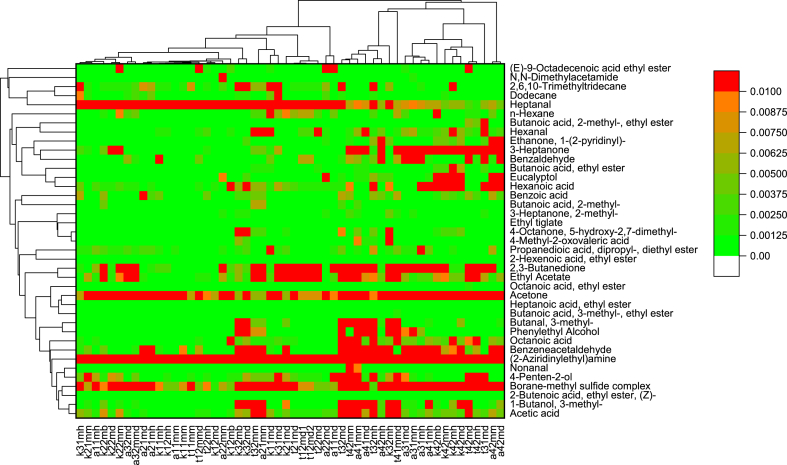
Heat map of camel milk volatile organic compounds concentrations (μL mL^−1^) and clusters issued from cluster analysis of the samples (in column) and of the different VOC (in row).

A total of 39 volatile aroma compounds were identified in the 54 experimental milk samples. The heat map with cluster analysis is presented in Fig. 1. Majority of VOCs were present in small concentrations, while acetone and (2-Aziridinylethyl) amine and to a lesser degree heptanal and borane methyl sulfide complex had high concentrations in almost all samples of camel milk.

**Fig. 2:**
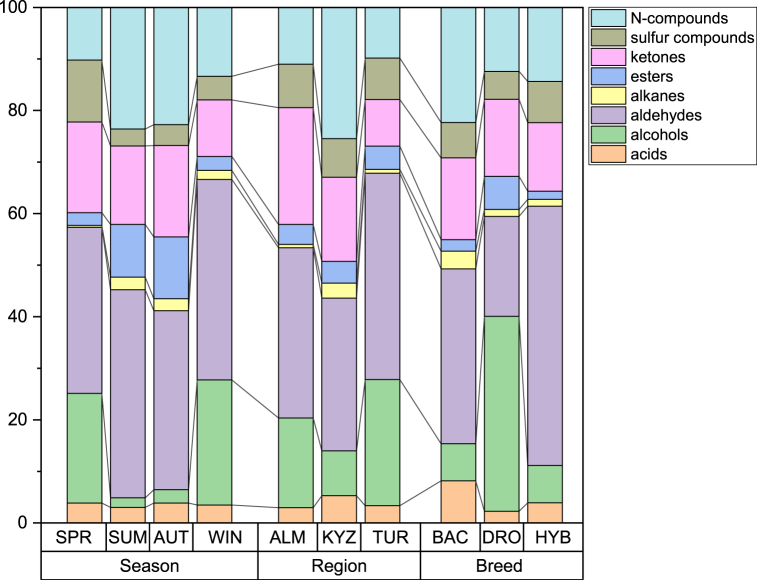
Average composition of camel milk samples by groups of VOCs (%). Difference of profiles by season, region, and camel breed.

To facilitate analysis and interpretation, all VOC molecules were grouped by type of compounds. The groups of these compounds were aldehydes (6), alcohols (4), alkane (3), ketones (6), esters (11), sulphur compounds (1), nitrogen compounds (2) and volatile acids (6). Fig. 2 represents different VOC group profiles according to region, season, and breed.

Camel milk contained higher proportion of aldehydes and alcohols in samples from the Turkistan region. Summer and autumn samples were similar, while spring samples contained higher proportion of ketones and sulphur compounds than winter samples. Samples differed by breed also, as samples from hybrid camels had the highest proportion of aldehydes. Dromedary samples had highest proportion of alcohols, while Bactrian samples contained highest proportion of acids and nitrogen compounds.

**Fig. 3:**
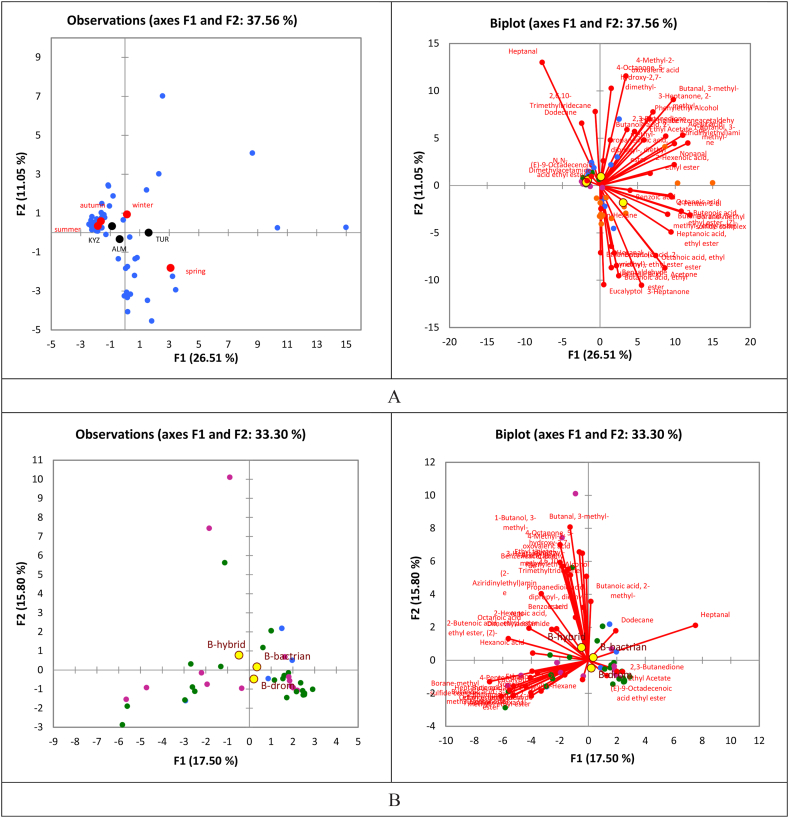
PCA plots regarding the projection of the milk samples on the main factorial plan (F1*F2) (left graphs) and the links with the volatile aroma compounds identified from camel milk on the same plan (right graphs): (A) Region and season centroids, (B) breed centroids as supplementary variables.

Overall PCA analysis shows that majority of compounds had low concentrations in summer and autumn seasons, while winter and spring samples have higher concentrations of VOCs (Fig. 3). Similarly, Kyzylorda region is close to summer and autumn samples, which makes it have lower concentrations of most of the VOCs than Almaty and Turkistan samples. Because the breed centroids are spread near the center of the PCA, the difference is small, especially given that we only had four samples of Bactrian milk.

AHC of milk samples revealed 4 groups. Chi square test of these groups with region, season and breed showed that the groups are significantly (p < 0.05) related to season of samples. The test also grouped summer and autumn samples together, while spring samples were present in 2 other groups, winter samples were scattered across all 4 groups.

**Fig. 4:**
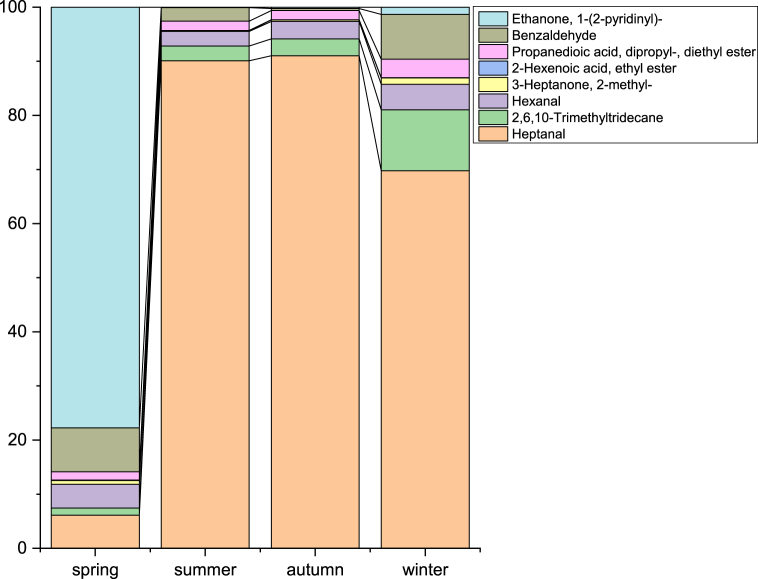
Average composition of discriminant VOCs (identified by discriminant factorial analysis) in camel milk according to the four seasons.

The discriminant analysis results (Table 2) revealed a greater number of discriminating molecules in seasons with a higher proportion of well-classified samples, highlighting a significant distinction, particularly between spring and other seasons. Spring samples were notably characterized by the lowest concentration of Heptanal, identified as the primary discriminating compound by season (Fig. 4). In terms of regional variations, Ethyl acetate, Butanoic acid, and Heptanone were identified as the key contributors to the differences observed. When considering breed differences, only two molecules, heptanal and hexanoic acid, emerged as discriminators, albeit with a lower percentage of well-classified samples. The iterative validation test achieved on 90 % of the data has shown a high stability of the results with similar percentage of well-classed.

**Table 2:** Results of discriminant analysis of volatile organic compounds in camel milk and shubat samples: list of discriminating VOCs, percentage of correctness (well-classed percentage) in the whole sample and after validation.

Groups	Milk - Season	Milk – Region	Milk – Breed	Shubat – Season	Shubat – Region
VOCs	Hexanal/3-Heptanone, 2-methyl-/Heptanal/Benzaldehyde/Ethanone, 1-(2-pyridinyl)-/2-Hexenoic acid, ethyl ester/2,6,10-Trimethyltridecane/Propanedioic acid, dipropyl-, diethyl ester	Ethyl Acetate/Butanoic acid, 2-methyl-, ethyl ester/3-Heptanone	Heptanal/Hexanoic acid	Heptanal	2,3-Butanediol, [R-(R*,R*)]-/Phenylethyl Alcohol/(E)-9-Octadecenoic acid ethyl ester
% correctness in confusion matrix	75.69	58.88	37.72	50	85
% correctness in confusion matrix for cross-validation results:	60.3	56.5	22.47	45	70

### 3.2 Shubat

**Fig. 5:**
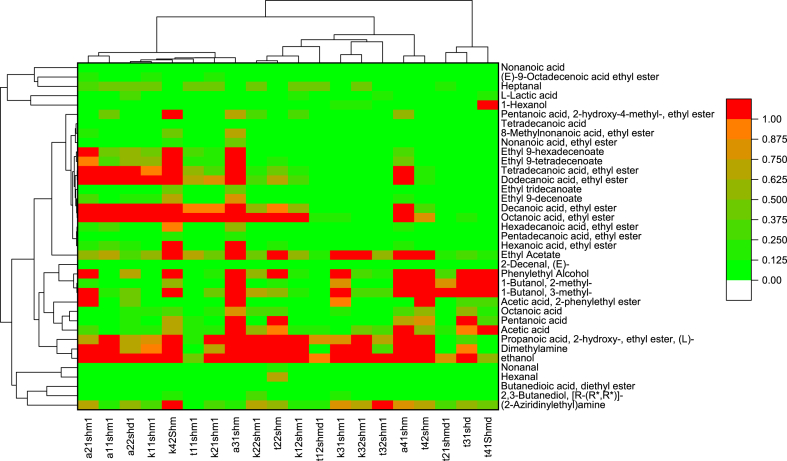
Heat map of shubat volatile organic compounds concentrations (μL mL^−1^) and clusters issued from cluster analysis of the shubat samples (in column) and of the different VOC (in row).

The utilization of bulk milk from various camel types in *shubat* production has led to the absence of distinctly identifiable *shubat* derived from Bactrian or hybrid camels. Consequently, this hindered the possibility of conducting breed-specific analyses on *shubat*. A total of 37 VOCs were identified in the experimental 20 *shubat* samples. A heat map VOCs with cluster analysis is presented in Fig. 5. *Shubat's* aroma is primarily composed of ethanol, dimethylamine, propanoic acid, octanoic acid, and ethyl acetate, as identified by VOC analysis. It should be noted that in heat map color scale of *shubat* samples is 10 times higher than in camel milk samples.

**Fig. 6:**
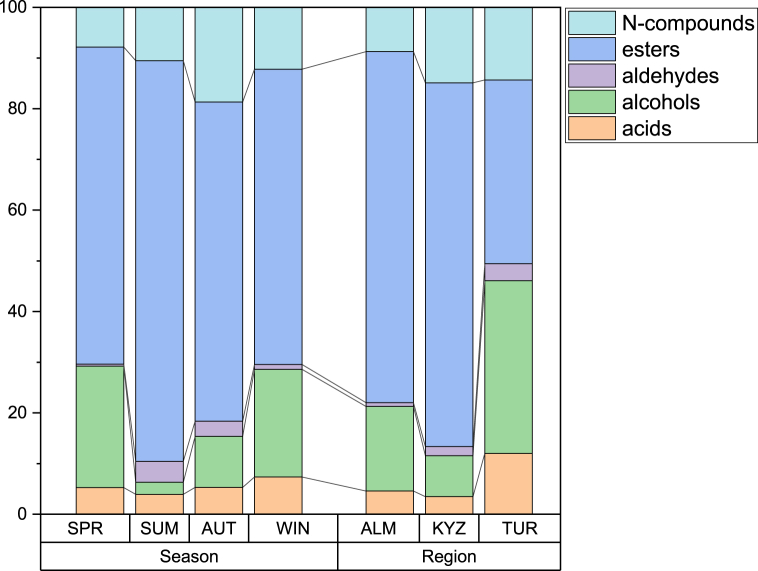
Average composition of shubat samples by groups of VOCs (%). Difference of profiles by season and region.

The groups of VOCs were aldehydes (4), alcohols (6), esters (19), nitrogen compounds (2) and volatile acids (6). In comparison with milk samples the strength of VOCs was higher in *shubat* and esters being the main group in aroma. Fig. 6 represents different VOC group profiles according to region and season.

Like camel milk *shubat* from Turkistan differed from Almaty and Kyzylorda samples, by having lowest proportion of esters and having highest proportion of alcohols and acids. Seasonally spring and winter samples show similarity and have highest alcohol proportions, while summer samples have highest proportion of esters, autumn samples have highest proportion of nitrogen compounds.

**Fig. 7:**
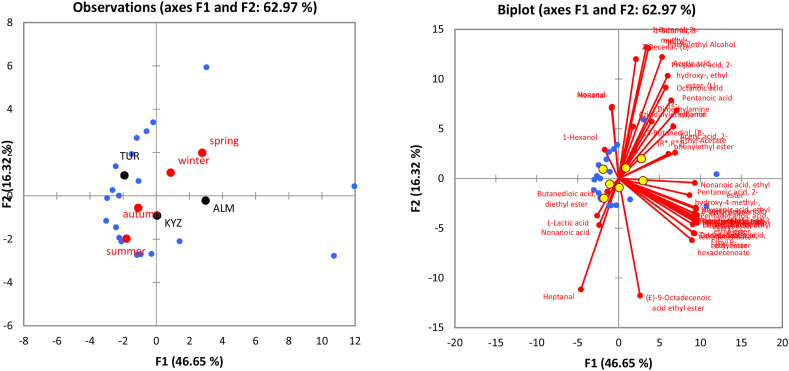
PCA plots regarding the projection of the shubat samples on the main factorial plan (F1*F2) (left graphs) and the links with the volatile aroma compounds identified from shubat on the same plan (right graphs); Region and season centroids were projected as supplementary variables.

The PCA plot again illustrates that majority of VOCs have higher concentrations in winter and spring season (Fig. 7). Similarly, centroid of Almaty is located towards most of VOCs, which makes it higher in VOCs concentrations than other regions.

AHC of *shubat* samples revealed 4 groups. Chi square test of these groups with region and season showed that the groups are significantly (p < 0.05) related to region of samples. The test also differentiates Turkistan samples from other regions.

**Fig. 8:**
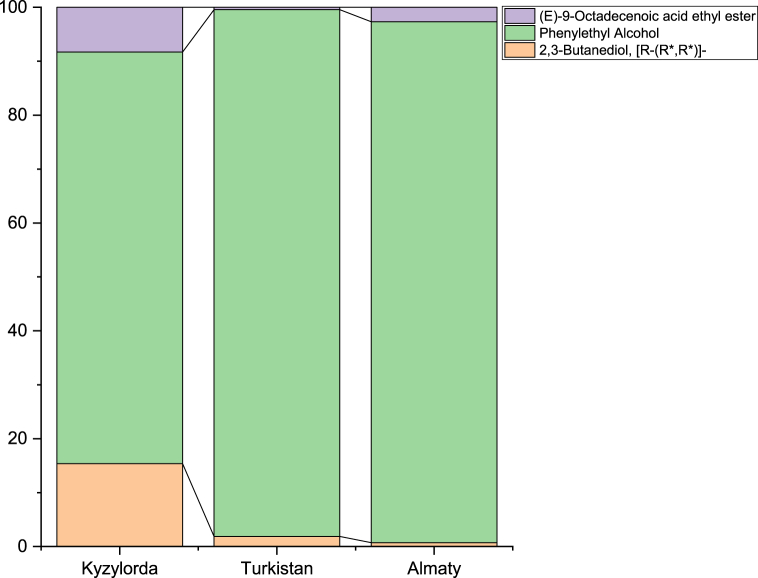
Average composition of discriminant VOCs (identified by discriminant factorial analysis) in shubat from three regions of Kazakhstan.

Key discriminating VOCs for *shubat* samples are also presented in Table 2. Heptanal emerges as a prominent indicator of seasonality, whereas Butenadiol, Phenylethyl alcohol, and Octadecenoic acid demonstrate strong discriminative capabilities for regional variations, successfully classifying 85 % of *shubat* samples. It is noteworthy that *shubat* samples from the Kyzylorda region exhibited a higher proportion of Butanediol and Octadecenoic acid compared to samples from other regions (Fig. 8).

## 4 Discussion

### 4.1 Camel milk

In the analysis of camel milk VOCs groups, elevated levels of aldehydes and alcohols suggest that, despite the precautions taken to transport and store samples in cold containers prior to analysis, some samples have undergone spontaneous fermentation. According to Meng, Chen, Sun, Li, Chen, Fang and Chen [12] alcohols, acids, esters and aldehydes increase with fermentation in mare milk.

Cluster analysis and chi^2^ test showed that season plays greater role than region or breed for milk samples. Seasonal effect could be linked to complex causes. Firstly, it could be attributed to dilution effect as the peak of lactation occurs in summer [13]. Additionally, difference in forage could be the reason. According to Baımukanov and Meredov [14] camels during spring season in Kazakhstan mainly consume ephemeral vegetation (Common daisy: *Leucanthemum vulgare*, Feather grass: *Stipa pennata* L., Besser Blue grass: *Poa versicolor*, Slender blue grass: *Poa attenuata*), while with the onset of the dry period in summer, camels start to consume drought-tolerant vegetation such as shrubs, subshrubs, diverse legume species, and halophytes (Devil's guts: *Convōlvulus arvēnsis*, Camelthorn: *Alhagi maurorum*, Couch grass: *Elytrígia répens*, Reed grass: *Calamagróstis epigéjos*, Ribbon grass: *Phalaris arundinacea*, Korshinsk pea shrub: *Caragana korshinskii*). During the autumn and winter seasons due to the significant reduction in grass availability, camels rely on thistles, subshrubs, shrubs, wormwood, and camel thorn as their primary source of nutrition (Camelthorn: *Alhagi maurorum*, Common saltwort: *Salsola tragus* L, Pallas saltwort: *Climacoptera lanata*, Pamirian winterfat: *Krascheninnikovia ceratoides*). Moreover, the camels are spending more time in-door in winter when the weather conditions do not allow out-door grazing.

Furthermore, the main discriminating VOC, heptanal was found to be coming from lipid oxidation [15]. Low concentration of heptanal in spring samples could mean high antioxidant concentration, which is confirmed by study of grass-finished beef where spring samples contained higher antioxidant concentrations [16].

### 4.2 Shubat

Various regions around the world produce fermented camel milk under different names, such as garris in Sudan, suusac in Kenya, and khoormog in Mongolia [17]. Despite the prevalence of these products, there is currently no detailed analysis of the VOC profiles associated with them. The distinctiveness of these fermented camel milk products is challenging to comprehend without such analyses. Conducting VOC analysis will provide an objective tool to facilitate a more effective and precise description of these diverse products in the future.

The observed variations in *shubat*, both regionally and seasonally, could potentially arise from differences in the composition of the camel milk used. However, seasonal differences were less pronounced compared to regional variations, contrasting with the camel milk samples. This suggests that the variations may also be attributed to the presence of diverse microbial communities that initiate the spontaneous milk fermentation process, consequently influencing the aromatic profile of *shubat*.

It is important to note that the microbial communities involved in *shubat* fermentation tend to remain consistent across seasons, as farmers traditionally use the *shubat* from previous fermentation as a starter. This consistency in microbial composition contributes to the relatively minor seasonal differences in *shubat* aroma.

In contrast, regional variations in *shubat* aroma are more pronounced. Traditional dairy products have distinct microflora that is based not only on production methods, but also on the ecological locations where they are produced [18]. In a previous study, lactic bacteria profiles in raw and fermented camel milk was investigated showing a wide regional difference linked to the environmental microbiota and variability in practices and equipment used for fermentation process [19]. As a result, this leads to variations in the fermentation process and the resulting aroma profile of *shubat*. This observation aligns with the findings of Mu, Yang and Yuan [20] who demonstrated that fermented mare milk from different regions exhibits distinct microbial compositions and consequently, varying taste and aroma profiles.

### 4.3 Groups of VOCs

Six and four aldehydes were identified in the experimental camel milk and *shubat* respectively. Transamination or Strecker degradation can contribute to the formation of aldehydes during the degradation of amino acids [21]. Among aldehydes Hexanal, Heptanal and Nonanal were found in both milk and *shubat*. These aldehydes are recognized for their contribution to undesirable aroma in cow milk [22]. Even though aldehydes are main compounds in milk, only little amount can be found in *shubat*, which is explained by transitory nature of aldehydes as they are rapidly reduced to alcohols or oxidized to acids [23].

Alcohols identified in the studied milk and *shubat* can be biosynthesized from amino acids, fatty acids or via glycolysis [24]. All three alcohols of camel milk (4-Penten-2-ol, 3-methyl-1-Butanol and Phenylethyl Alcohol) had high contribution to first principal component (PC1). In *shubat* among 6 alcohols only ethanol had high contribution to PC1, while 3-methyl-1-Butanol, 2-methyl-1-Butanol and Phenylethyl Alcohol contributed highly to second principal component (PC2). 2+3-methyl-1-Butanols were reported to have Alcoholic, green odor, while Phenylethyl Alcohol pleasant aroma of rose flower [23]. In the study of Ning, Fu-Ping, Hai-Tao, Si-Yuan, Chen, Zhen-Yang and Bao-Guo [8] the main group of VOCs in *shubat* were alcohols with 3-Methyl-1-butanol and Phenylethyl Alcohol having highest concentration.

Esters are a product of esterification between acids and alcohol. They can also form from lactose fermentation or amino acid catabolism [23]. Esters are described as having pleasant sweet and fruity notes [25]. The high presence of esters in *shubat* bears resemblance to the maturation process of cheese, wherein a notable increase in ester concentration was observed during the advanced stages of ripening of camel milk cheese [7].

Ethyl Acetate was the one of the main esters in both milk and *shubat*, however it was absent in shubat studied by Ning, Fu-Ping, Hai-Tao, Si-Yuan, Chen, Zhen-Yang and Bao-Guo [8]. They reported that among esters 2-Hydroxy-propanoic acid ethyl ester had highest concentration, which is absent in our samples of *shubat*.

Three nitrogen containing compounds were found in our samples namely, (2-Aziridinylethyl) amine in both milk and *shubat*, N,N-Dimethylacetamide in milk and Dimethylamine in *shubat* only. (2-Aziridinylethyl)-amine contributed highly to PC1 in camel milk, other's contributions were low. (2-Aziridinylethyl)amine was found in different types of plants [26,27], which could be the reason of presence in milk and shubat. None of abovementioned nitrogen containing compounds were found in other studies of shubat and camel milk cheese [7,8].

Six acids were discovered in both milk and *shubat*, but only two were shared: acetic acid and octanoic acid. These acids made a significant contribution to PC1 in camel milk and *shubat*. Pentanoic acid was also a significant contributor to PC1 in *shubat*. In our *shubat* samples, we identified nearly all acids reported in the study by Ning, Fu-Ping, Hai-Tao, Si-Yuan, Chen, Zhen-Yang and Bao-Guo [8], except for l-lactic acid, which they did not detect. Hailu, Hansen, Seifu, Eshetu, Petersen, Lametsch, Rattray and Ipsen [7] reported that acetic acid in camel milk cheese increases with ripening time. It is likely that the fermentation and maturation processes of *shubat* and camel milk cheese diverge due to their distinct microbiota. This difference arises from the different structures of the medium, as well as different fermentations conditions.

Alkanes, ketones and sulphur compounds were only found in camel milk. None of alkanes had high contribution to PC1 or PC2. Among ketones Acetone and 2-methyl-3-Heptanone, had high contribution to PC1. Acetone was also found in camel milk cheese [7], however it has unpleasant flavor [28]. Additionally, acetone represents two-thirds of total VOC concentration in cow milk [29]. Other authors report that acetone is detectable in the milk of cows experiencing ketosis [30]. 2-methyl-3-Heptanone is one of the trace organic compounds that impart flavor and odor to natural water [31].

## 5 Conclusion

Overall, our investigation offers a comprehensive description of VOCs and unveils notable variations in the VOC profiles of both camel milk and *shubat*. In general, camel milk samples contained higher concentrations of aldehydes, ketones, and alcohols, whereas aroma profile of *shubat* samples were dominated by esters. Milk samples primarily distinguished themselves by season, likely due to factors like seasonal variations in feeding (forage) and fluctuations during the lactation period, primarily associated with dilution effects. On the other hand, *shubat* exhibited regional variations driven primarily by diverse microbial communities within the production areas (farms). Further in-depth investigations are required to definitively characterize the regional composition of microbial communities in camel milk and *shubat* and their precise impact on VOC profiles. By integrating VOC analysis with microbiota studies, we can optimize production practices, ensure consistent quality, and contribute to the development of novel fermented beverages with unique sensory properties. Moreover, the VOC profile holds promise for standardizing camel milk production based on geographical indications and enhancing the overall quality of various dairy products derived from camel milk. Presently, descriptions of aroma in camel milk and other traditional fermented products are limited and subjective, relying solely on verbal descriptors. Integrating VOC analysis can provide a more rigorous and standardized approach, enhancing quality assessment and regulatory frameworks in the camel milk industry and related dairy sectors.

## Data availability

The authors confirm that the data supporting the findings of this study are available within the article [and/or] its supplementary materials.

## CRediT authorship contribution statement

**Zauresh Bilal:** Writing – original draft, Investigation, Formal analysis, Data curation. **Askar Kondybayev:** Writing – review & editing, Investigation, Formal analysis. **Aikerim Ospanova:** Writing – review & editing, Investigation, Formal analysis. **Helene Tormo:** Writing – review & editing, Supervision, Project administration. **Shynar Akhmetsadykova:** Writing – review & editing, Supervision, Project administration. **Farida Amutova:** Writing – review & editing, Investigation, Formal analysis. **Bernard Faye:** Writing – review & editing, Supervision, Formal analysis. **Gaukhar Konuspayeva:** Writing – review & editing, Supervision, Project administration, Conceptualization.

## Declaration of competing interest

The authors declare that they have no known competing financial interests or personal relationships that could have appeared to influence the work reported in this paper.
