# Supplementary material for: Volatile organic compounds of camel milk and shubat across Kazakhstan's regions, seasons, and breeds
Source: Heliyon. 2024 Jul 29;10(15):e35365. doi: 10.1016/j.heliyon.2024.e35365 (PMC11336635; doi:10.1016/j.heliyon.2024.e35365)
Supplement: Multimedia component 2 [file mmc2.docx]

Table S1

| Season | Region | milk | | | | *shubat* | | | |
| --- | --- | --- | --- | --- | --- | --- | --- | --- | --- |
|  |  | pH 1 | SD | pH2 | SD | pH 1 | SD | pH2 | SD |
| summer | Almaty | 6.7 | 0.2 | 6.4 | 0.1 | 4.2 | 0.0 | 3.9 | 0.0 |
|  | Kyzylorda | 6.8 | 0.5 | 6.2 | 0.2 | 5.6 | 0.0 | 3.4 | 0.1 |
|  | Turkistan | 6.5 | 0.4 | 6.2 | 0.8 | 5.5 | 0.1 | 3.8 | 0.4 |
| autumn | Almaty | 6.3 | 0.2 | 6.2 | 0.3 | 4.9 | 0.9 | 4.1 | 0.2 |
|  | Kyzylorda | 6.5 | 0.1 | 6.2 | 0.1 | 4.9 | 0.1 | 4.0 | 0.0 |
|  | Turkistan | 6.4 | 0.5 | 6.2 | 0.5 | 5.1 | 0.2 | 4.0 | 0.3 |
| winter | Almaty | 7.0 | 0.5 | 7.0 | 0.5 | 5.1 | 0.1 | 4.8 | 0.2 |
|  | Kyzylorda | 7.0 | 0.0 | 6.7 | 0.2 | 5.2 | 0.3 | 4.7 | 0.3 |
|  | Turkistan | 6.2 | 0.2 | 5.5 | 0.3 | 5.7 | 0.0 | 5.0 | 0.2 |
| spring | Almaty | 6.7 | 0.4 | 5.9 | 0.3 | 4.9 | 0.0 | 4.0 | 0.0 |
|  | Kyzylorda | 6.9 | 0.3 | 6.3 | 0.0 | 5.6 | 0.0 | 4.0 | 0.0 |
|  | Turkistan | 6.8 | 0.5 | 6.4 | 0.3 | 5.4 | 0.0 | 4.2 | 0.2 |

pH 1 - value immediately after milking

pH 2 - value after transporting samples to the laboratory
